# A Distinct Perisynaptic Glial Cell Type Forms Tripartite Neuromuscular Synapses in the *Drosophila* Adult

**DOI:** 10.1371/journal.pone.0129957

**Published:** 2015-06-08

**Authors:** Alexandra L. Strauss, Fumiko Kawasaki, Richard W. Ordway

**Affiliations:** Department of Biology and Center for Molecular and Cellular Neuroscience, The Pennsylvania State University, University Park, Pennsylvania, United States of America; Columbia University, UNITED STATES

## Abstract

Previous studies of *Drosophila* flight muscle neuromuscular synapses have revealed their tripartite architecture and established an attractive experimental model for genetic analysis of glial function in synaptic transmission. Here we extend these findings by defining a new *Drosophila* glial cell type, designated peripheral perisynaptic glia (PPG), which resides in the periphery and interacts specifically with fine motor axon branches forming neuromuscular synapses. Identification and specific labeling of PPG was achieved through cell type-specific RNAi-mediated knockdown (KD) of a glial marker, Glutamine Synthetase 2 (GS2). In addition, comparison among different *Drosophila* neuromuscular synapse models from adult and larval developmental stages indicated the presence of tripartite synapses on several different muscle types in the adult. In contrast, PPG appear to be absent from larval body wall neuromuscular synapses, which do not exhibit a tripartite architecture but rather are imbedded in the muscle plasma membrane. Evolutionary conservation of tripartite synapse architecture and peripheral perisynaptic glia in vertebrates and *Drosophila* suggests ancient and conserved roles for glia-synapse interactions in synaptic transmission.

## Introduction

A common synaptic architecture in vertebrates involves assembly of presynaptic and postsynaptic elements with glial processes to form tripartite (three-part) synapses. Previous work has established important roles for glia-synapse interactions in synaptic development and function (reviewed in [1–3]). Studies of vertebrate neuromuscular synapses, for example, have shown that specialized peripheral glia called Perisynaptic Schwann Cells (PSCs) contribute to tripartite synapse structure and function [4–6]. Recent work on *Drosophila* neuromuscular synapses, specifically those of the Dorsal Longitudinal Flight Muscles (DLM) in the adult, established the presence of tripartite synapses and functional glia-synapse interactions in an invertebrate [7, 8]. Thus *Drosophila* offers a unique experimental model in which powerful genetic approaches may be applied to the study of glutamatergic tripartite synapses which are accessible in the periphery.

Despite this progress, the origin of glial cell processes which participate in tripartite DLM neuromuscular synapses, and thus the potential for selective genetic manipulation of these glial elements, has not been determined. Among several known types of peripheral glia in *Drosophila* [9, 10], including those which ensheath peripheral axons, none has been implicated in glia-synapse interactions. In the absence of a cell type-specific marker which labels perisynaptic glial processes, it is not known whether a distinctive type of glial cell contributes to tripartite neuromuscular synapses. Here we have utilized cell type-specific KD of a glial marker, GS2, as a novel approach to identify and specifically label perisynaptic glia. Moreover, electrophysiological studies of GS2 KD synapses extend previous genetic and functional analysis of GS2 [11], a *Drosophila* enzyme homologous to glial cytoplasmic Glutamine Synthetases implicated in neural function [12]. The present study provides further characterization of *Drosophila* tripartite neuromuscular synapses by defining a new type of peripheral glial cell which provides synaptic glial processes. In addition, this work establishes that glia-synapse interactions are a common feature of several different neuromuscular synapses of the *Drosophila* adult.

## Results

**Fig 1:**
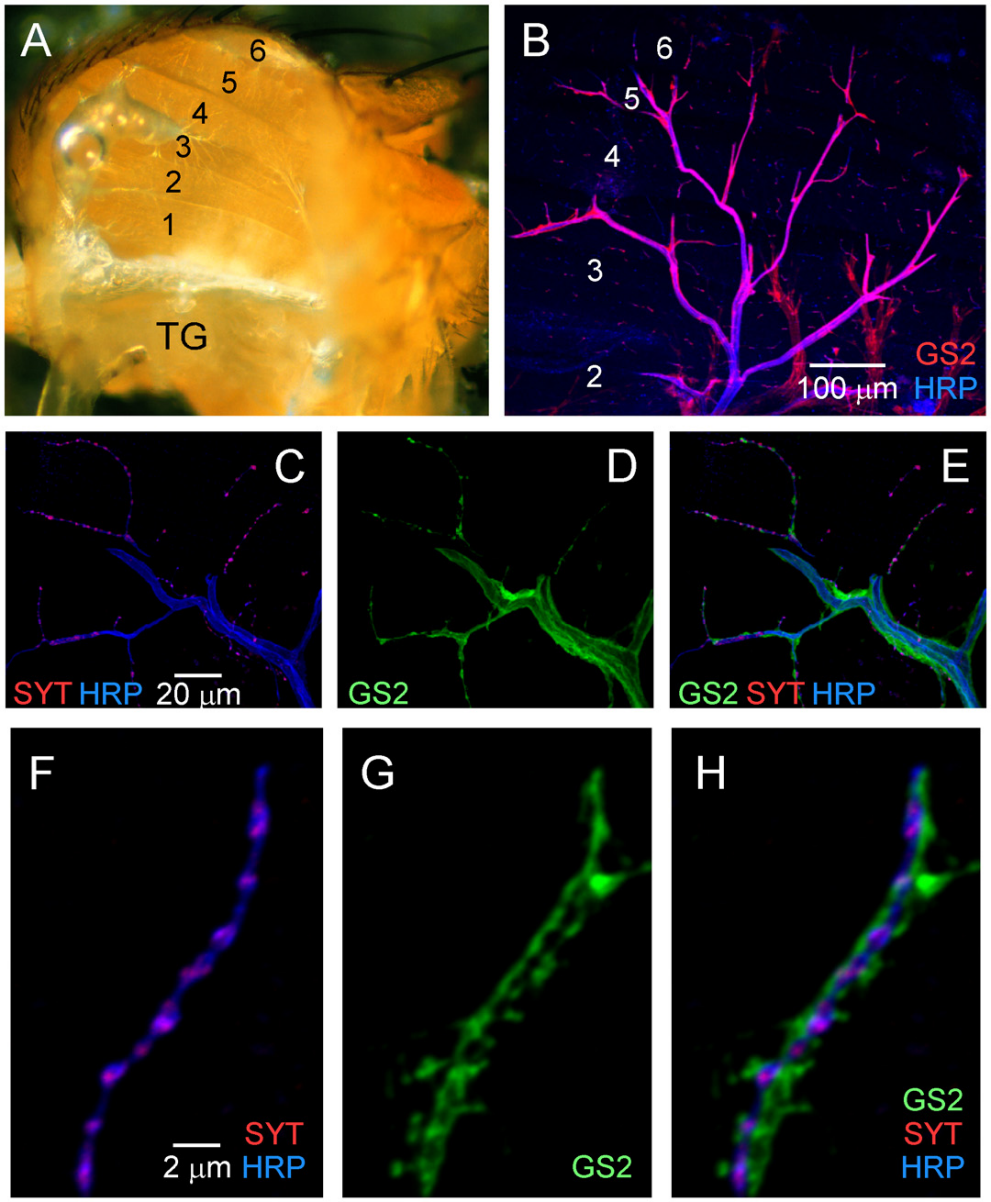
Organization of tripartite DLM neuromuscular synapses. (**A**) A lateral view of the dissected thorax showing a stack of six DLM fibers (numbered). Each fiber is innervated by a motor axon projecting from the thoracic ganglion (TG) through the Posterior Dorsal Mesothoracic Nerve (PDMN). (**B**) Confocal immunofluorescence image showing the distribution of PDMN branches (blue) and associated glia (red) over the surface of a DLM stack. Anti-HRP labels the neuronal plasma membrane and anti-GS2 labels glia. (**C-H**) Confocal immunofluorescence images of DLM neuromuscular synapses. Triple labeling with anti-HRP, anti-GS2 and anti-SYNAPTOTAGMIN (SYT, a synaptic vesicle marker). Fine terminal axon branches make synaptic contacts on the muscle (**C,F**) in association with glial processes (**D,G**) to form tripartite neuromuscular synapses (**E,H**).

The DLM neuromuscular synapse preparation (Fig 1A) includes six DLM muscle fibers innervated by five motor axons [13, 14] which exit the CNS within the Posterior Dorsal Mesothoracic Nerve (PDMN). Main branches of the PDMN project to the surface of the DLMs as indicated by neuronal and glial markers (Fig 1B) and motor axons branch extensively over the muscle surface in close association with glia (Fig 1C–1E). Fine terminal axon branches make synaptic contacts on the muscle (Fig 1C) and interact with glial processes (Fig 1D and 1E) to form tripartite neuromuscular synapses (Fig 1F–1H, S1 Fig and [7]). However, the cellular organization of glia at DLM neuromuscular synapses has not been defined. Although cell type-specific genetic approaches provide the potential for *in vivo* analysis of glial function, the use of glial molecular markers, such as GS2 [15–17] and the glutamate transporter, dEAAT1 [18], cannot distinguish whether synaptic glial processes are contributed by a distinct cell type. Further analysis took advantage of the GAL4-UAS system [19] to generate a cell type-specific marker which labels perisynaptic glia.

**Fig 2:**
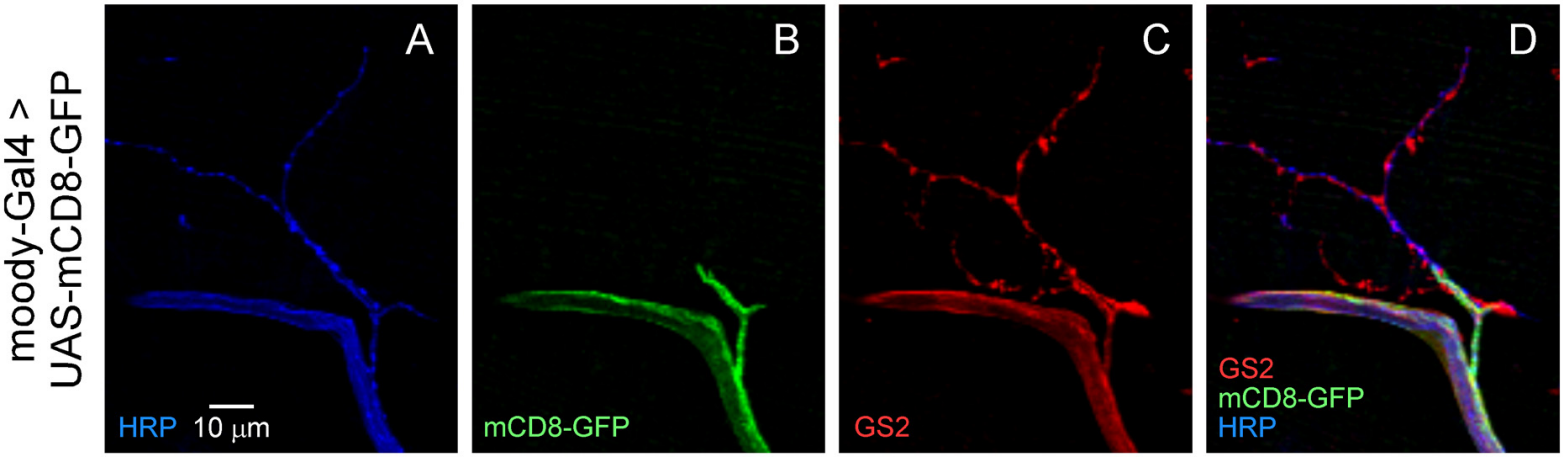
*moody*-GAL4 drives expression in nerve-associated glia forming the blood-brain barrier but not perisynaptic glial processes. Confocal immunofluorescence and native GFP fluorescence images of DLM neuromuscular synapses. Expression of membrane-associated GFP (mCD8-GFP) using the *moody*-GAL4 driver is restricted to nerve-associated glia, in contrast to the pattern of GS2 which includes perisynaptic glial processes.

In initial studies, different "driver" transgenic lines, which express the yeast GAL4 transcription factor in a cell type-specific manner, were crossed to flies carrying a transgene for expression of membrane-targeted GFP under the control of a GAL4-responsive Upstream Activation Sequence (UAS-*mCD8*-*GFP*). A screen of previously established glial GAL4 driver lines identified none that selectively labeled a distinct glial cell type in proximity to synapses. However, several lines which express in peripheral glia failed to mark synaptic glial processes labeled by GS2. One example is *moody*-GAL4 [20] which drives expression in subperineurial glial cells, a subtype of glia that forms the blood-brain barrier in peripheral nerves [21, 22]. Using this GAL4 driver, GFP expression is not observed in glial processes associated with neuromuscular synapses (Fig 2) and thus subperineurial and perisynaptic glia represent distinct cell types. These findings suggested that specific labeling of perisynaptic glia might be accomplished by using RNAi to restrict expression of a glial marker to this cell type. As described in the following text, this was achieved by selective KD of *Gs2* in *moody*-positive subperineurial glia which do not contribute to tripartite synapses.

**Fig 3:**
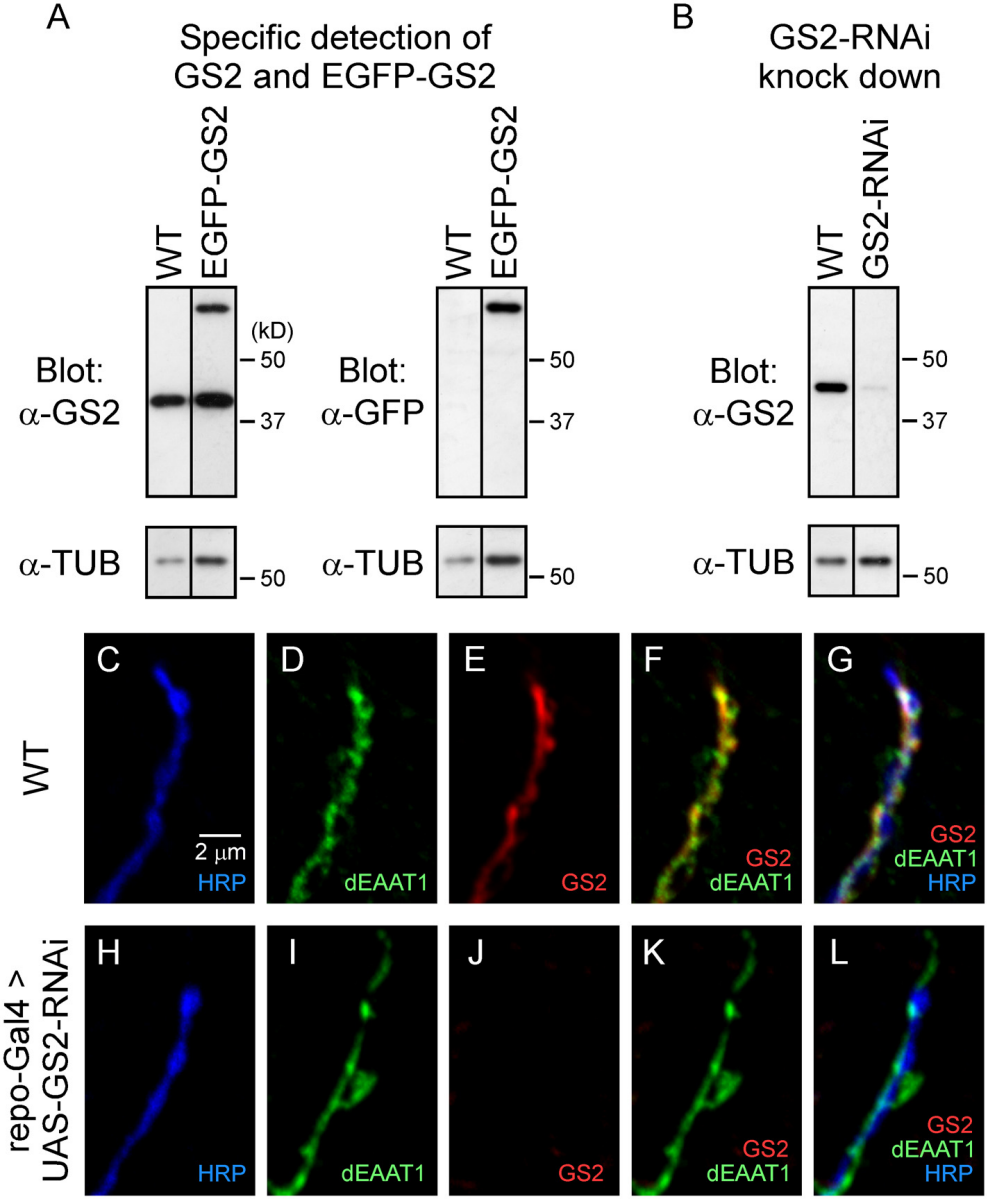
Efficient GS2 KD by RNAi. (**A**) Western analysis of head homogenates from wild-type (WT) flies and those expressing a UAS-*EGFP*-*Gs2* transgene (EGFP-GS2) under the control of the pan-glial driver, *repo*-GAL4. Specific detection of GS2 was confirmed using anti-GS2 and anti-GFP antibodies. Anti-GS2 recognized both endogenous GS2 and the larger EGFP-GS2 fusion protein. (**B**) Western analysis of WT flies and those expressing a UAS-GS2-RNAi transgene under the control of *repo*-GAL4. Glial-specific KD of GS2 produced a marked reduction in GS2 detected in head homogenates. (**C-L**) Confocal immunofluorescence images showing glial expression of GS2 at DLM neuromuscular synapses (**C-G**) and a marked reduction after glial-specific KD of GS2 (**H-L**).

No transgenic lines were available for RNAi-mediated KD of *Gs2* and so UAS lines for expression of a *Gs2*-RNAi transgene were generated. Expression of the *Gs2*-RNAi transgene using a pan-glial driver, *repo*-GAL4 [23], resulted in marked KD of *Gs2* as assessed by Western and immunocytochemistry. Westerns were carried out using an antibody which was generated against sheep Glutamine Synthetase and is referred to here as anti-GS2. The ability of this antibody to detect *Drosophila* GS2 [24] was further confirmed through transgenic expression of a GFP-GS2 fusion protein (Fig 3A). Western analysis of flies in which a UAS-*Gs2*-RNAi transgene was expressed using the pan-glial *repo*-GAL4 driver indicated that glial-specific KD of *Gs2* produced a marked reduction in GS2 levels in head homogenates (Fig 3B). Consistent with this observation, parallel immunocytochemical analysis confirmed KD of *Gs2* in glial processes at DLM neuromuscular synapses (Fig 3C–3L). Notably, flies were viable after pan-glial KD of *Gs2* and exhibited no obvious impairment in motor behavior. Furthermore, initial studies of DLM neuromuscular synaptic function indicated that both the initial excitatory postsynaptic current (EPSC) amplitude and the EPSC amplitude after 60 seconds of 20 Hz train stimulation were similar to the corresponding wild-type values (S2 Fig). In light of the marked reduction in GS2 levels after KD, and the fact that *Gs2* appears to encode the only cytoplasmic Glutamine Synthetase in *Drosophila* [15], these observations indicate this enzyme is not required in glia to support the basic functional properties of DLM neuromuscular synapses. Future studies will address whether GS2 is necessary to sustain glutamate release during more prolonged periods of synaptic activity as indicated in recent studies of Glutamine Synthetase at mammalian synapses [25].

**Fig 4:**
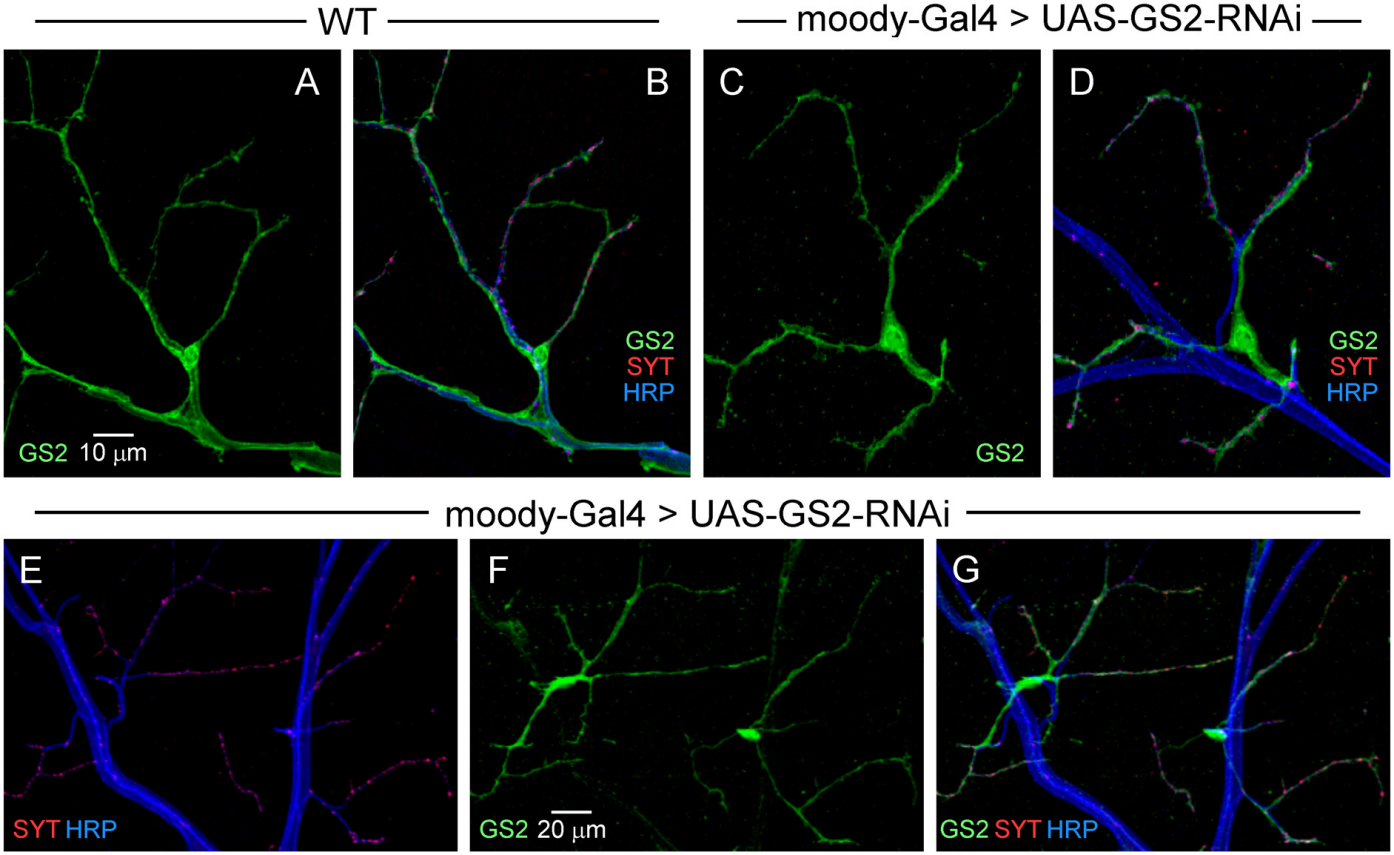
GS2 KD in *moody*-positive subperineurial glia reveals selective labeling of perisynaptic glial cells. Confocal immunofluorescence images of DLM neuromuscular synapses as described in Fig 1. In contrast to the distribution of GS2 observed in wild-type (**A, B**), GS2 was restricted to perisynaptic glia following *Gs2*-RNAi KD using the *moody*-GAL4 driver (**C,D**). (**E-G**) The spatial distribution of perisynaptic glial cells over the muscle surface indicates that each occupies a distinct territory in which it makes contacts with local synapses.

**Fig 5:**
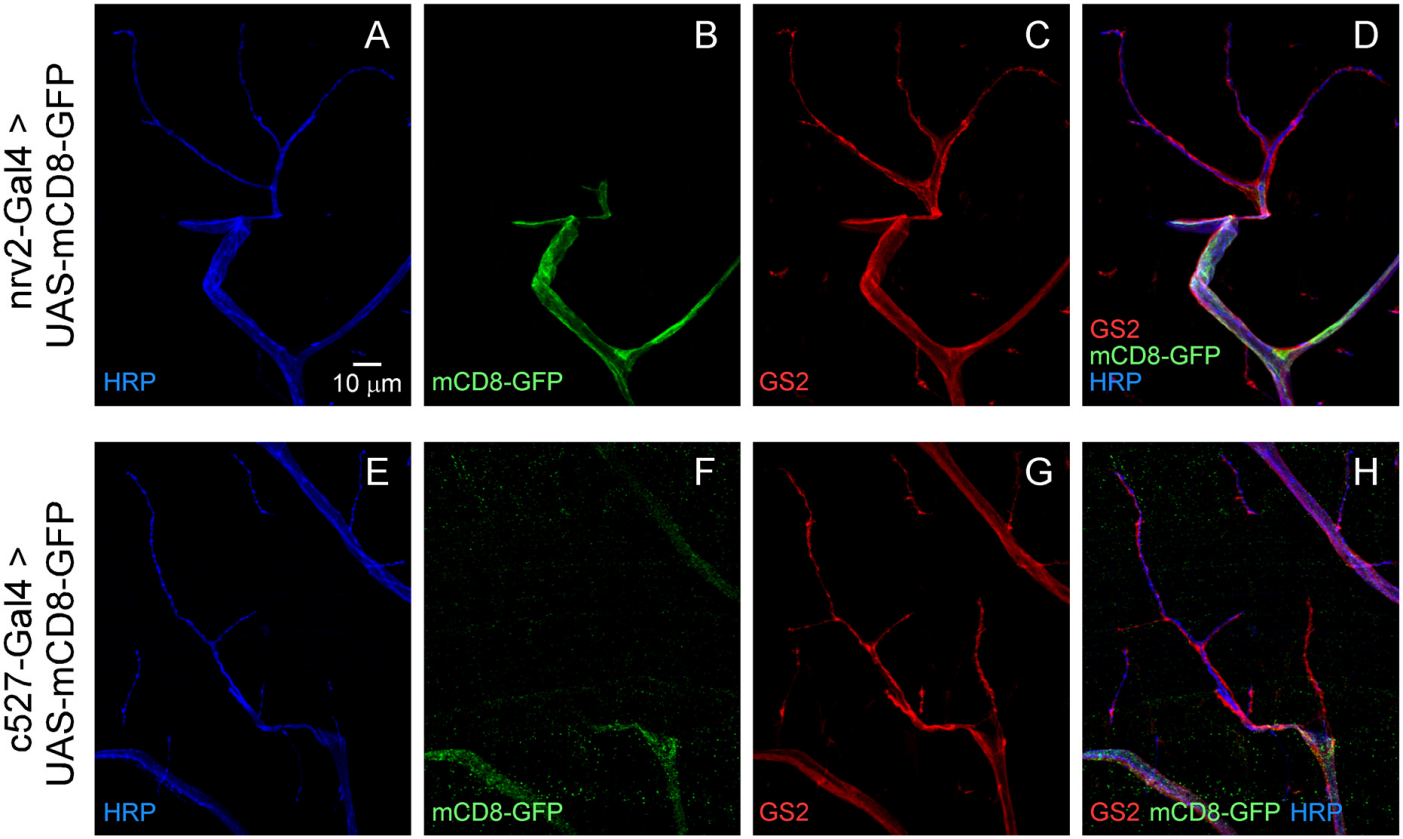
Perisynaptic glial cells are distinct from previously characterized wrapping and perineurial glia. Confocal immunofluorescence and native GFP fluorescence images of DLM neuromuscular synapses. Expression of membrane-associated GFP (mCD8-GFP) using drivers specific for wrapping (*nrv2*-GAL4, **A-D**) or perineurial (*c527*-Gal4, **E-H**) glia are restricted to the nerve trunk and major branches, in contrast to the pattern of GS2 which includes perisynaptic glial processes.

On the basis of the preceding studies, efforts to develop a specific marker for perisynaptic glia were pursued through cell type-specific KD of *Gs2* using the *moody*-GAL4 driver. This approach was effective in that the GS2 signal was largely eliminated from the nerve trunk and major branches but remained in glia cells which participate in tripartite synapses (Fig 4A–4D). These studies reveal that perisynaptic glial processes are provided by a subset of glial cells with distinctive properties. Note that their cell bodies are located in the periphery in proximity to terminal branches of the motor axons (Fig 4D). At the point where fine motor axon branches project from the nerve to form synaptic contacts on the muscle, perisynaptic glial processes extend to and selectively interact with synapses (Fig 4D). Finally, these studies reveal a spatial distribution of perisynaptic glia over the muscle surface indicating that each occupies a distinct territory and contacts local synapses (Fig 4E–4G). Although these perisynaptic glial cells share several molecular markers with other glia, including the pan-glial nuclear protein, REPO (S3 Fig), they are distinct from three types of peripheral glia characterized previously [9, 10] as assessed using cell type-specific GAL4 driver lines. In addition to studies of *moody*-positive subperineurial glia (Fig 2), wrapping and perineurial glia were marked using the *nrv2*-GAL4 [26, 27] and *c527*-GAL4 [28] drivers, respectively. Like *moody*-GAL4, both of these drivers marked glia in the peripheral nerve but neither labeled perisynaptic glia (Fig 5). These findings indicate that tripartite synapses are formed by a distinct type of perisynaptic glial cell which we designate peripheral perisynaptic glia (PPG).

**Fig 6:**
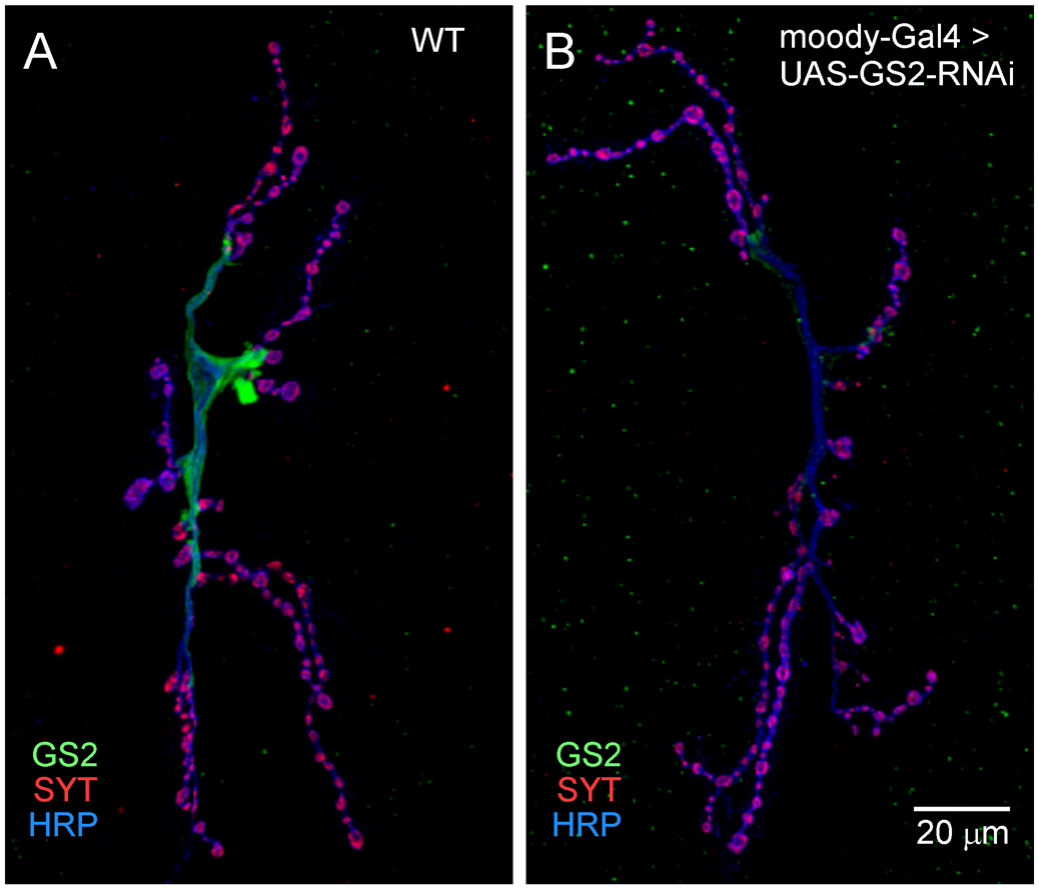
Absence of perisynaptic glia at larval body wall neuromuscular synapses. Confocal immunofluorescence images of larval neuromuscular synapses as described in Fig 1 (**A**) In WT, GS2 is associated with non-synaptic regions of motor axons but does not extend to fine axon branches which form synapses. (**B**) *Gs2*-RNAi KD using *moody*-GAL4 largely eliminated the GS2 signal at larval neuromuscular synapses and did not reveal the presence of a perisynaptic glial cell type.

To examine the extent to which PPGs and a tripartite architecture are general features of *Drosophila* neuromuscular synapses, similar approaches were used to examine other neuromuscular synapses present at the larval and adult stages. The larval body wall neuromuscular synapse has been an important and widely used model for genetic analysis of synapse development and function [29–31]. Notably, this synapse differs from that of the adult DLM with regard to both its functional [32] and morphological properties [33]. Distinctive morphological features of the larval neuromuscular synapse include large presynaptic boutons which contain a densely packed array of presynaptic active zones and are embedded within an elaborate infolding of the muscle plasma membrane called the subsynaptic reticulum (SSR) [34]. Consistent with previous work [35], immunocytochemical analysis of larval neuromuscular synapses indicated that the GS2 marker labels glia which are associated with non-synaptic regions of motor axons but do not extend to fine axon branches forming synapses (Fig 6A). KD of *Gs2* in subperineurial glia using *moody*-GAL4 largely eliminated the GS2 signal at larval neuromuscular synapses and did not reveal the presence of a perisynaptic glial cell type (Fig 6B).

**Fig 7:**
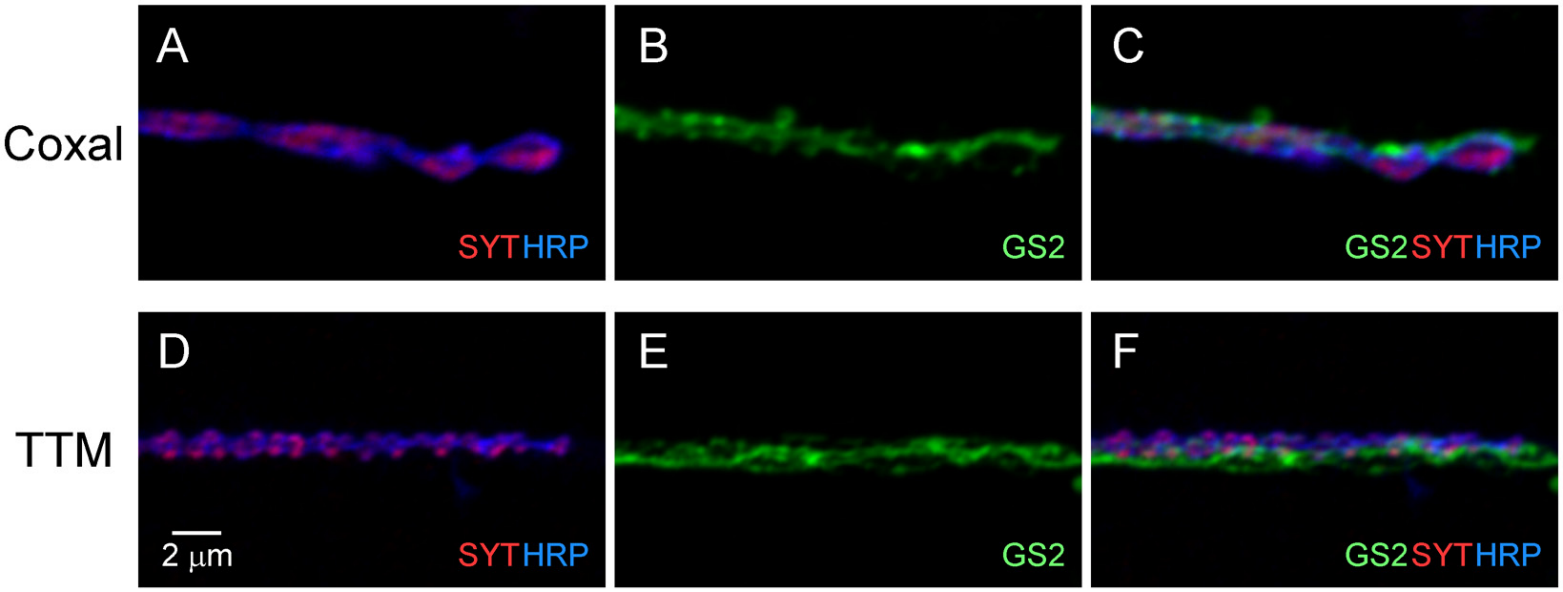
Tripartite interaction of perisynaptic glial processes with pre- and postsynaptic elements is a shared feature of several different adult neuromuscular synapses. Confocal immunofluorescence images of neuromuscular synapses as described in Fig 1. Fine terminal axon branches making synaptic contacts on coxal muscles in the leg (**A-C**) and the tergotrochanteral muscle (TTM) in the thorax (**D-F**) exhibit close association with glial processes as observed at DLM neuromuscular synapses.

**Fig 8:**
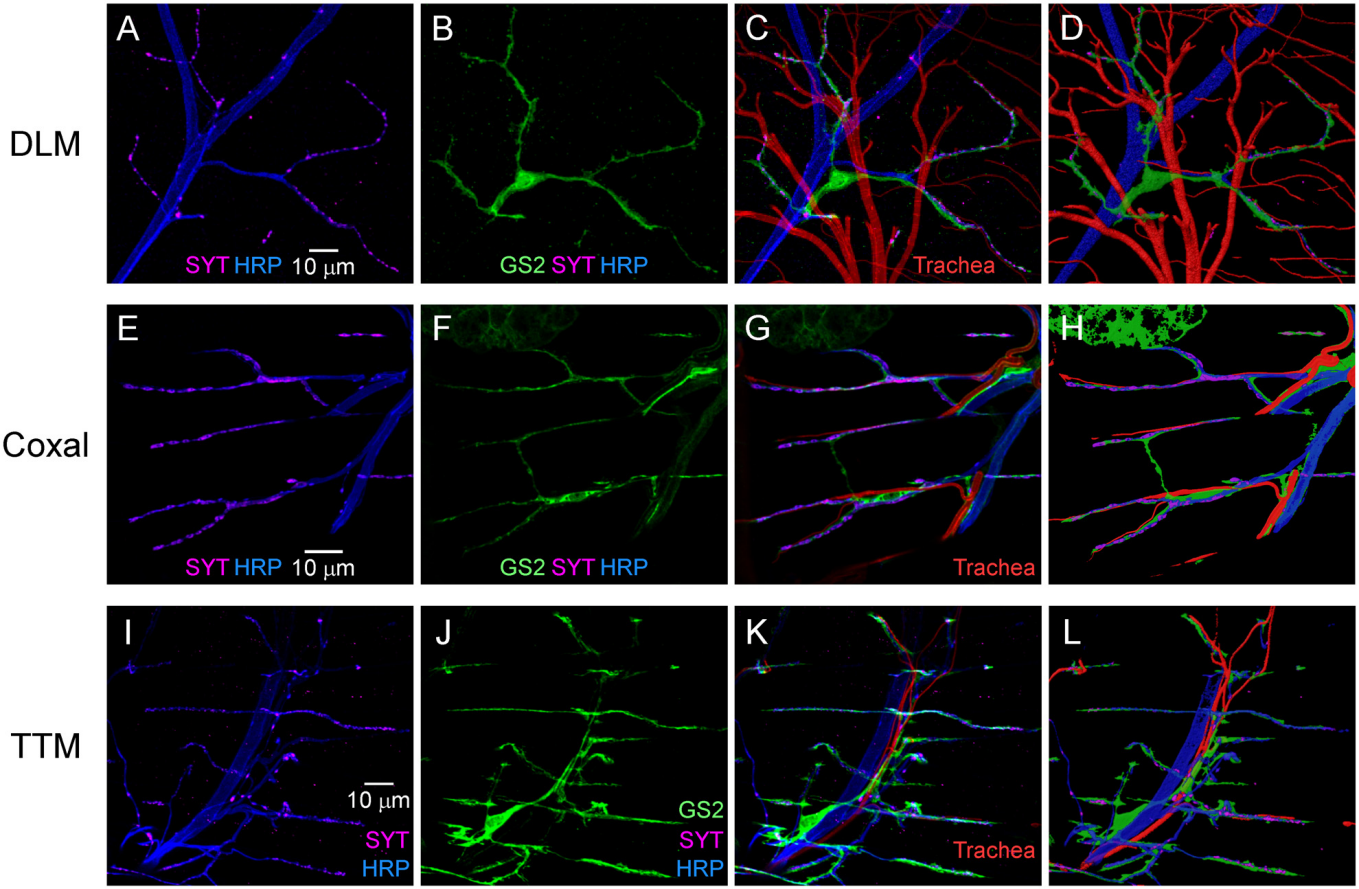
Spacial organization of cell interactions at different adult neuromuscular synapses. Confocal immunofluorescence images of neuromuscular synapses on DLM (**A-D**), coxal muscles (**E-H**) and TTM (**I-L**) as described in Fig 1. GS2 was restricted to PPG following *Gs2*-RNAi KD using the *moody*-GAL4 driver. (**D,H,L**) Three dimensional model of tripartite synapse organization including interactions with tracheal vessels (generated with the Software package, Imaris).

Finally, a broader examination of adult neuromuscular synapses indicated the tripartite architecture of the DLM synapse is shared with those of two adult muscle types which are morphologically and functionally distinct from the DLMs: the coxal muscles (Fig 7A–7C) and the tergotrochanteral muscles (TTM) (Fig 7D–7F). Moreover, the organization of cell interactions associated with these different tripartite neuromuscular synapses exhibits interesting similarities as well as distinctive features. All three types exhibit a high degree of synaptic coverage by PPG processes (Fig 8), suggesting a critical role for tripartite synapse architecture at adult neuromuscular synapses. However, clear differences were observed in the interactions of tripartite synapses with tracheal vessels mediating gas exchange. As reported previously [7], tripartite DLM neuromuscular synapses contact fine tracheal vessels which are present within a dense network of trachea distributed throughout the muscle (Fig 8C and 8D). In contrast, the more restricted distribution of trachea in coxal muscles resembles that of neuronal and glial processes and all three elements are in close association (Fig 8G and 8H). TTM synapses exhibit a third variation in which tracheal vessels provide less coverage of synapses and associate primarily with major nerve branches (Fig 8K and 8L). Taken together, the preceding findings reveal a predominant tripartite morphology as well as distinctive spatial organizations among neuromuscular synapses in the *Drosophila* adult.

## Discussion

The results reported here identify a new type of peripheral glial cell in *Drosophila*, named PPG, and demonstrate that a distinct perisynaptic glial cell type contributes to tripartite neuromuscular synapses of the adult. A genetic approach distinguished PPG from other peripheral glia on the basis of their unique combination of glial molecular markers as well as the peripheral location of their cell bodies and their characteristic morphological interactions at neuromuscular synapses. Identification of PPG further defines the cellular organization of tripartite neuromuscular synapses in *Drosophila* and enhances this experimental model for genetic analysis of synapse-glia interactions.

Identification of *Drosophila* PPG invites comparison to peripheral glia contributing to vertebrate tripartite neuromuscular synapses. These are referred to as Perisynaptic Schwann Cells (PSCs) and have been characterized most extensively at neuromuscular synapses of the frog and mouse [4, 6]. For both PSCs and PPGs, the cell body is located peripherally in proximity to neuromuscular synapses and each cell occupies a distinct territory on the muscle surface ([36] and Fig 4E–4G). Furthermore, the processes of both cell types interact specifically with fine branches of motor axons which make synaptic contacts on the muscle. Finally, both PSC and PPG exhibit synaptic activity-induced calcium transients [5, 7]. One key difference between PSCs and PPGs is that the former contributes to cholinergic and the latter to glutamatergic neuromuscular synapses. Accordingly, PPG express GS2 and other conserved markers of tripartite glutamatergic synapses, including glutamate transporters which contribute to synaptic function [7]. Thus analysis of PPG function in *Drosophila* represents a unique experimental model in which the molecular mechanisms of glial function in glutamatergic synaptic transmission may be investigated through genetic analysis at accessible peripheral synapses.

Our findings indicate that a tripartite morphology involving direct glia-synapse interactions is common among several glutamatergic neuromuscular synapses of the *Drosophila* adult. In contrast, these interactions are absent at body wall neuromuscular synapses of the larva. This observation is interesting in light of the distinctive functional properties of larval body wall and adult DLM neuromuscular synapses [32] and their different developmental and functional roles in the organism. Synapse-glia interactions may support the refined functional properties of mature synapses in the adult, which participate in relatively complex physiological processes such as flight and adult motor activity. In contrast, larval body wall neuromuscular synapses support simpler functions such as crawling and feeding during a period of remarkably rapid development and growth. It is of interest to consider whether the distinctive morphology of larval body wall neuromuscular synapses, which are imbedded in the muscle SSR, may provide glial-like support for these synapses through a different mechanism. As more information emerges about the direct interactions of glia and synapses in the *Drosophila* CNS, it may not be surprising to find tripartite synaptic morphologies at both the adult and larval stages.

Finally, the presence of a common tripartite architecture among several *Drosophila* adult neuromuscular synapses suggests it is of widespread importance in motor function. With regard to the evolution of glia [37], it is of interest to note that tripartite synapses [7] and peripheral perisynaptic glia were either present in a common ancestor of arthropods and vertebrates or rather emerged independently by convergent evolution. Future studies of other invertebrate synapses are expected to further define this evolutionary relationship and the extent to which a tripartite morphology is a shared feature of synapses throughout the animal kingdom.

## Materials and Methods

### *Drosophila* strains

The *moody*-GAL4 and *c527*-GAL4 driver transgenic lines were kindly provided by Dr. Marc Freeman (University of Massachusetts Medical School, Worcester, MA) and Dr. Christian Klämbt (Institute of Neurobiology, University of Münster, Germany), respectively. Flies obtained from the Bloomington Stock Center included the *repo*-GAL4 driver (stock #7415), the *nrv2*-GAL4 driver (stock #6797) and the UAS-*mCD8*-*GFP* (stock #32186) transgenic lines. The UAS-*EGFP*-*Gs2* and UAS-*Gs2*-RNAi transgenic lines were generated in this study (see **UAS-transgenic lines**). Wild-type flies were *Canton S*. Stocks and crosses were cultured on a conventional cornmeal-molasses-yeast medium at 20–22°C.

UAS-transgenic lines were generated for expression of EGFP-GS2, in which EGFP is fused to the GS2 N-terminus, as well as a dsRNA “hairpin” for RNAi KD of GS2 (*Gs2*-RNAi). To generate the UAS-*EGFP*-*Gs2* transgene, the *Gs2* ORF was amplified from a cDNA clone (clone ID: GH03580; GenBank accession number: BT099804) obtained from the *Drosophila* Genomics Research Center. A construct encoding *EGFP*-*Gs2* was cloned into the Not I and Kpn I sites of the P element transformation vector, pUAST [19]. To generate the UAS-*Gs2*-RNAi transgene, two identical PCR products corresponding to the initial 828bp of the *Gs2* ORF were cloned into the pGEM-WIZ vector [38] as inverted repeats in a head-to-head orientation. The resulting construct was excised with Bgl II and Xba I and shuttled into pUAST. Transgenic flies were generated as described previously [39].

Western blotting and immunocytochemistry were carried out essentially as described previously ([40] and [33], respectively). Western blotting was performed with the following primary antibodies (listed with dilution, source): Mouse monoclonal anti-Glutamine Synthetase Clone GS-6 (referred to here as anti-GS2) (1:1,000, Millipore, Darmstadt, Germany, # MAB302), rabbit anti-GFP (1:1,000, Life Technologies, Grand Island, NY, #A6455) and mouse monoclonal anti-acetylated Tubulin Clone 6-11B-1 (1:2,000,000, Sigma, St. Louis, MO, #T6793). Immunocytochemistry was performed with the following primary antibodies: Alexa 647-conjugated goat anti-Horseradish Peroxidase (HRP) (1:200, Jackson Immunoresearch Laboratories, West Grove, PA, #123-605-021), rabbit anti-Synaptotagmin (SYT) (1:5,000, generously provided by Dr. Noreen Reist, Colorado State University, Fort Collins, CO), rabbit anti-*Drosophila* Excitatory Amino Acid Transporter 1 (dEAAT1) (1:2,000, generously provided by Dr. Serge Birman, Developmental Biology Institute of Marseille, France), mouse monoclonal anti-REPO Clone 8D12 (1:20, Developmental Studies Hybridoma Bank, Iowa City, IA) and anti-GS2 (1:500). These studies employed Alexa Fluor secondary antibodies (1:200, Life Technologies, Grand Island, NY) and trachea were visualized by autofluorescence using excitation with a 405 nm laser. Immunocytochemical studies of GFP typically involved imaging native GFP fluorescence. However, in the case of the c527-GAL4 driver which produced relatively weak expression, a rabbit anti-GFP antibody was used to enhance the signal (1:1000, Life Technologies, Grand Island, NY, #A6455).

Voltage-clamp recording of EPSCs at DLM neuromuscular synapses was carried out essentially as described previously [41]. Microsoft (Seattle, WA) Excel was utilized to analyze numerical data and generate graphs. Data values are presented as mean ± SEM. Statistical significance was determined using the two-tailed Student's t test and significance was assigned to comparisons for which *p* ≤ 0.05.

## Supporting Information

S1 Fig Tripartite morphology of DLM neuromuscular synapses. Confocal immunofluorescence images of DLM neuromuscular synapses. (**A**) Anti-HRP labels the neuronal plasma membrane and anti-DPAK labels postsynaptic densities closely apposed to presynaptic active zones. (**B**) Anti-GS2 labels glial processes and (**C**) reveals their close association with axons and synapses. Note that synaptic contacts with the postsynaptic membrane are not covered by glial processes.
(TIF)

S2 Fig Synaptic transmission at DLM neuromuscular synapses is preserved after pan-glial KD of GS2. Two-electrode voltage-clamp recordings of synaptic currents from DLM neuromuscular synapses. (**A**) Scaled and superimposed single EPSCs indicate similar EPSC waveforms at wild-type (WT) and *Gs2*-RNAi KD synapses. For the *Gs2*-RNAi KD, the pan-glial *repo*-GAL4 driver was used to express the UAS-*Gs2*-RNAi transgene. The initial EPSC amplitudes for WT and GS2-RNAi KD were 1.88 ± 0.15 μA (n = 4) and 1.71 ± 0.18 μA (n = 4), respectively, and were not significantly different (p = 0.28). (**B**) *Gs2*-RNAi KD synapses exhibit wild-type short-term depression during sustained train stimulation at 20 Hz. Peak EPSC amplitudes were normalized to the initial amplitude and plotted as a function of stimulus number. After 60 seconds of train stimulation, the EPSC amplitudes for WT and Gs2-RNAi KD were reduced to 26.0 ± 4.56% (n = 4) and 33.0 ± 2.46% (n = 4), respectively. These values were not significantly different (p = 0.07), although there may be slightly less synaptic depression at *Gs2*-RNAi KD synapses. Error bars represent the S.E.M.
(TIF)

S3 Fig PPG express the pan-glial nuclear marker, REPO. Confocal immunofluorescence and native GFP fluorescence images of DLM neuromuscular synapses. Experiments to examine REPO expression in PPG using the available mouse monoclonal anti-REPO antibody could not utilize the mouse monoclonal anti-GS2 antibody to selectively label PPG as in Fig 4. Rather, an alternative strategy employed *moody*-GAL4 to drive mCD8-GFP expression and mark ensheathing subperineurial glia as in Fig 2. After labeling with the rabbit polyclonal anti-dEAAT1 antibody, PPG can be distinguished as those glia expressing dEAAT1 but not GFP (A-C). Labeling with the anti-REPO antibody was imaged in the same channel with autofluorescence from the tracheal system (D-F, trachea, REPO). Anti-REPO marked the nuclei of ensheathing glia (arrowheads) as well as PPG (arrow). Note that these results are consistent with loss of GS2 signal in PPG after GS2 KD using the repo-Gal4 driver (Fig 3C–3L).
(TIF)

## References

[1] Araque A, Carmignoto G, Haydon PG, Oliet SH, Robitaille R, Volterra A. Gliotransmitters travel in time and space. Neuron. 2014;81(4):728–39. Epub 2014/02/25. 10.1016/j.neuron.2014.02.007 24559669 PMC4107238

[2] Nedergaard M, Verkhratsky A. Artifact versus reality--how astrocytes contribute to synaptic events. Glia. 2012;60(7):1013–23. Epub 2012/01/10. 10.1002/glia.22288 22228580 PMC3340515

[3] Jackson FR, Haydon PG. Glial cell regulation of neurotransmission and behavior in *Drosophila* . Neuron Glia Biol. 2008;4:11–7. 10.1017/S1740925X09000027 18950546

[4] Sugiura Y, Lin W. Neuron-glia interactions: the roles of Schwann cells in neuromuscular synapse formation and function. Bioscience reports. 2011;31(5):295–302. Epub 2011/10/01. 10.1042/BSR20100107 21517783 PMC4573580

[5] Todd KJ, Darabid H, Robitaille R. Perisynaptic glia discriminate patterns of motor nerve activity and influence plasticity at the neuromuscular junction. J Neurosci. 2010;30:11870–82. 10.1523/JNEUROSCI.3165-10.2010 20810906 PMC6633406

[6] Feng Z, Ko CP. Neuronal glia interactions at the vertebrate neuromuscular junction. Curr Opin Pharmacol. 2007;7(3):316–24. Epub 2007/04/03. 10.1016/j.coph.2006.12.003 . 17400025

[7] Danjo R, Kawasaki F, Ordway RW. A tripartite synapse model in *Drosophila* . PLoS One. 2011;6:e17131 10.1371/journal.pone.0017131 21359186 PMC3040228

[8] Rival T, Soustelle L, Cattaert D, Strambi C, Iché M, Birman S. Physiological requirement for the glutamate transporter dEAAT1 at the adult *Drosophila* neuromuscular junction. J Neurobiol. 2006;66:1061–74. . 16838372 10.1002/neu.20270

[9] Hartenstein V. Morphological diversity and development of glia in Drosophila. Glia. 2011;59(9):1237–52. Epub 2011/03/26. 10.1002/glia.21162 21438012 PMC3950653

[10] Rodrigues F, Schmidt I, Klambt C. Comparing peripheral glial cell differentiation in Drosophila and vertebrates. Cell Mol Life Sci. 2011;68(1):55–69. Epub 2010/09/08. 10.1007/s00018-010-0512-6 . 20820850 PMC11114915

[11] Featherstone DE, Rushton E, Broadie K. Developmental regulation of glutamate receptor field size by nonvesicular glutamate release. Nat Neurosci. 2002;5(2):141–6. Epub 2001/12/26. 10.1038/nn789 . 11753421

[12] Rose CF, Verkhratsky A, Parpura V. Astrocyte glutamine synthetase: pivotal in health and disease. Biochem Soc Trans. 2013;41(6):1518–24. Epub 2013/11/22. 10.1042/BST20130237 . 24256247

[13] Sun Y-A, Wyman RJ. Neurons of the *Drosophila* giant fiber system; I. Dorsal longitudinal motor neurons. J Comp Neurol. 1997;387:157–66. 9331179

[14] Ikeda K, Koenig JH. Morphological identification of the motor neurons innervating the dorsal longitudinal flight muscle of *Drosophila melanogaster* . J Comp Neurol. 1988;273:436–44. 3145293 10.1002/cne.902730312

[15] Caizzi R, Bozzetti MP, Caggese C, Ritossa F. Homologous nuclear genes encode cytoplasmic and mitochondrial glutamine synthetase in Drosophila melanogaster. J Mol Biol. 1990;212(1):17–26. Epub 1990/03/05. 10.1016/0022-2836(90)90301-2 . 1969491

[16] Freeman MR, Delrow J, Kim J, Johnson E, Doe CQ. Unwrapping glial biology: *gcm* target genes regulating glial development, diversification, and function. Neuron. 2003;38:567–80. 12765609 10.1016/s0896-6273(03)00289-7

[17] Thomas GB, van Meyel DJ. The glycosyltransferase Fringe promotes Delta-Notch signaling between neurons and glia, and is required for subtype-specific glial gene expression. Development. 2007;134(3):591–600. Epub 2007/01/12. 10.1242/dev.02754 . 17215308

[18] Besson MT, Soustelle L, Birman S. Selective high-affinity transport of aspartate by a *Drosophila* homologue of the excitatory amino-acid transporters. Curr Biol. 2000;10:207–10. . 10704415 10.1016/s0960-9822(00)00339-0

[19] Brand AH, Perrimon N. Targeted gene expression as a means of altering cell fates and generating dominant phenotypes. Development. 1993;118:401–15. . 8223268 10.1242/dev.118.2.401

[20] Schwabe T, Bainton RJ, Fetter RD, Heberlein U, Gaul U. GPCR signaling is required for blood-brain barrier formation in drosophila. Cell. 2005;123(1):133–44. Epub 2005/10/11. 10.1016/j.cell.2005.08.037 . 16213218

[21] Stork T, Engelen D, Krudewig A, Silies M, Bainton RJ, Klambt C. Organization and function of the blood-brain barrier in Drosophila. J Neurosci. 2008;28(3):587–97. Epub 2008/01/18. 10.1523/JNEUROSCI.4367-07.2008 . 18199760 PMC6670337

[22] Bainton RJ, Tsai LT, Schwabe T, DeSalvo M, Gaul U, Heberlein U. moody encodes two GPCRs that regulate cocaine behaviors and blood-brain barrier permeability in Drosophila. Cell. 2005;123(1):145–56. Epub 2005/10/11. 10.1016/j.cell.2005.07.029 . 16213219

[23] Sepp KJ, Schulte J, Auld VJ. Peripheral glia direct axon guidance across the CNS/PNS transition zone. Dev Biol. 2001;238:47–63. 11783993 10.1006/dbio.2001.0411

[24] Sinakevitch I, Grau Y, Strausfeld NJ, Birman S. Dynamics of glutamatergic signaling in the mushroom body of young adult *Drosophila* . Neural Dev. 2010;5:10 10.1186/1749-8104-5-10 20370889 PMC3003247

[25] Tani H, Dulla CG, Farzampour Z, Taylor-Weiner A, Huguenard JR, Reimer RJ. A local glutamate-glutamine cycle sustains synaptic excitatory transmitter release. Neuron. 2014;81(4):888–900. Epub 2014/02/25. 10.1016/j.neuron.2013.12.026 24559677 PMC4001919

[26] Pereanu W, Shy D, Hartenstein V. Morphogenesis and proliferation of the larval brain glia in Drosophila. Dev Biol. 2005;283(1):191–203. Epub 2005/05/24. 10.1016/j.ydbio.2005.04.024 . 15907832

[27] Sun B, Xu P, Salvaterra PM. Dynamic visualization of nervous system in live Drosophila. Proc Natl Acad Sci U S A. 1999;96(18):10438–43. Epub 1999/09/01. 10468627 10.1073/pnas.96.18.10438 PMC17907

[28] Hummel T, Attix S, Gunning D, Zipursky SL. Temporal control of glial cell migration in the Drosophila eye requires gilgamesh, hedgehog, and eye specification genes. Neuron. 2002;33(2):193–203. Epub 2002/01/24. . 11804568 10.1016/s0896-6273(01)00581-5

[29] Hallermann S, Silver RA. Sustaining rapid vesicular release at active zones: potential roles for vesicle tethering. Trends Neurosci. 2013;36(3):185–94. Epub 2012/11/21. 10.1016/j.tins.2012.10.001 . 23164531 PMC4748400

[30] Menon KP, Carrillo RA, Zinn K. Development and plasticity of the Drosophila larval neuromuscular junction. Wiley interdisciplinary reviews Developmental biology. 2013;2(5):647–70. Epub 2013/09/10. 10.1002/wdev.108 24014452 PMC3767937

[31] Thomas U, Sigrist SJ. Glutamate receptors in synaptic assembly and plasticity: case studies on fly NMJs. Advances in experimental medicine and biology. 2012;970:3–28. Epub 2012/02/22. 10.1007/978-3-7091-0932-8_1 . 22351049

[32] Wu Y, Kawasaki F, Ordway RW. Properties of short-term synaptic depression at larval neuromuscular synapses in wild-type and temperature-sensitive paralytic mutants of *Drosophila* . J Neurophysiol. 2005;93:2396–405. . 15845998 10.1152/jn.01108.2004

[33] Kawasaki F, Zou B, Xu X, Ordway RW. Active zone localization of presynaptic calcium channels encoded by the *cacophony* . J Neurosci. 2004;24:282–5. . 14715960 10.1523/JNEUROSCI.3553-03.2004 PMC6729574

[34] Jan LY, Jan YN. Properties of the larval neuromuscular junction in *Drosophila melanogaster* . J Physiol. 1976;262(1):189–214. . 11339 10.1113/jphysiol.1976.sp011592 PMC1307637

[35] Sepp KJ, Schulte J, Auld VJ. Developmental dynamics of peripheral glia in *Drosophila melanogaster* . Glia. 2000;30:122–33. 10719354 10.1002/(sici)1098-1136(200004)30:2<122::aid-glia2>3.0.co;2-b

[36] Brill MS, Lichtman JW, Thompson W, Zuo Y, Misgeld T. Spatial constraints dictate glial territories at murine neuromuscular junctions. J Cell Biol. 2011;195(2):293–305. Epub 2011/10/19. 10.1083/jcb.201108005 22006952 PMC3198169

[37] Freeman MR, Rowitch DH. Evolving concepts of gliogenesis: a look way back and ahead to the next 25 years. Neuron. 2013;80(3):613–23. Epub 2013/11/05. 10.1016/j.neuron.2013.10.034 . 24183014 PMC5221505

[38] Bao S, Cagan R. Fast cloning inverted repeats for RNA interference. RNA. 2006;12(11):2020–4. Epub 2006/09/29. 10.1261/rna.258406 17005926 PMC1624905

[39] Kawasaki F, Collins SC, Ordway RW. Synaptic calcium channel function in *Drosophila*: Analysis and transformation rescue of temperature-sensitive paralytic and lethal mutations of *cacophony* . J Neurosci. 2002;22:5856–64. . 12122048 10.1523/JNEUROSCI.22-14-05856.2002 PMC6757926

[40] Kawasaki F, Iyer J, Posey LL, Sun CE, Mammen SE, Yan H, et al The DISABLED protein functions in CLATHRIN-mediated synaptic vesicle endocytosis and exoendocytic coupling at the active zone. Proc Natl Acad Sci USA. 2011;108:E222–9. 10.1073/pnas.1102231108 21606364 PMC3121831

[41] Kawasaki F, Ordway RW. Molecular mechanisms determining conserved properties of short-term synaptic depression revealed in NSF and SNAP-25 conditional mutants. Proc Natl Acad Sci USA. 2009;106:14658–63. 10.1073/pnas.0907144106 19706552 PMC2732793

