# Supplementary figures and images for: A Distinct Perisynaptic Glial Cell Type Forms Tripartite Neuromuscular Synapses in the Drosophila Adult
Source: PLoS One. 2015 Jun 8;10(6):e0129957. doi: 10.1371/journal.pone.0129957 (PMC4459971; doi:10.1371/journal.pone.0129957)

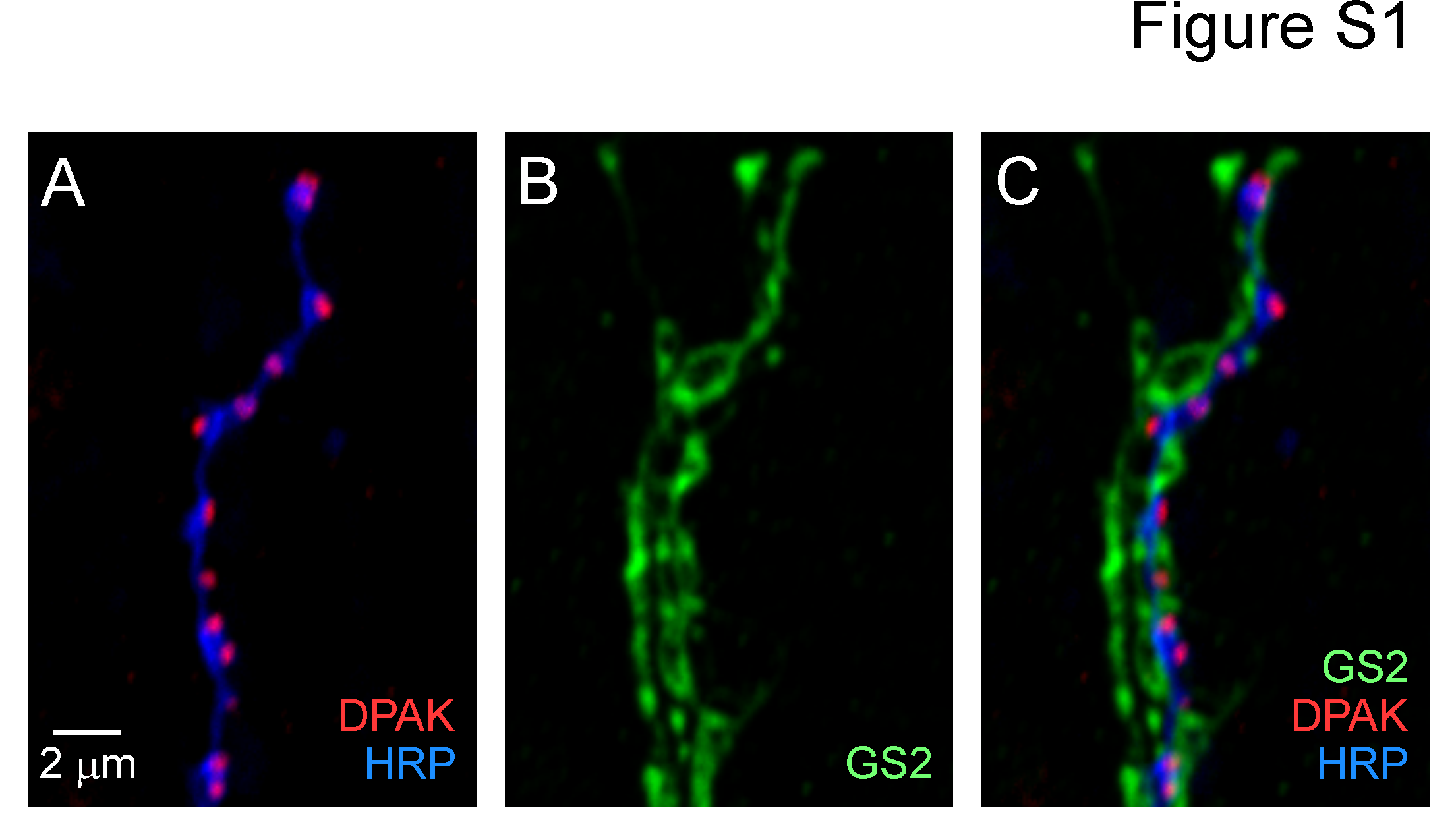

Supplement: S1 Fig — Confocal immunofluorescence images of DLM neuromuscular synapses. (A) Anti-HRP labels the neuronal plasma membrane and anti-DPAK labels postsynaptic densities closely apposed to presynaptic active zones. (B) Anti-GS2 labels glial processes and (C) reveals their close association with axons and synapses. Note that synaptic contacts with the postsynaptic membrane are not covered by glial processes. (TIF) [file pone.0129957.s001.tif]

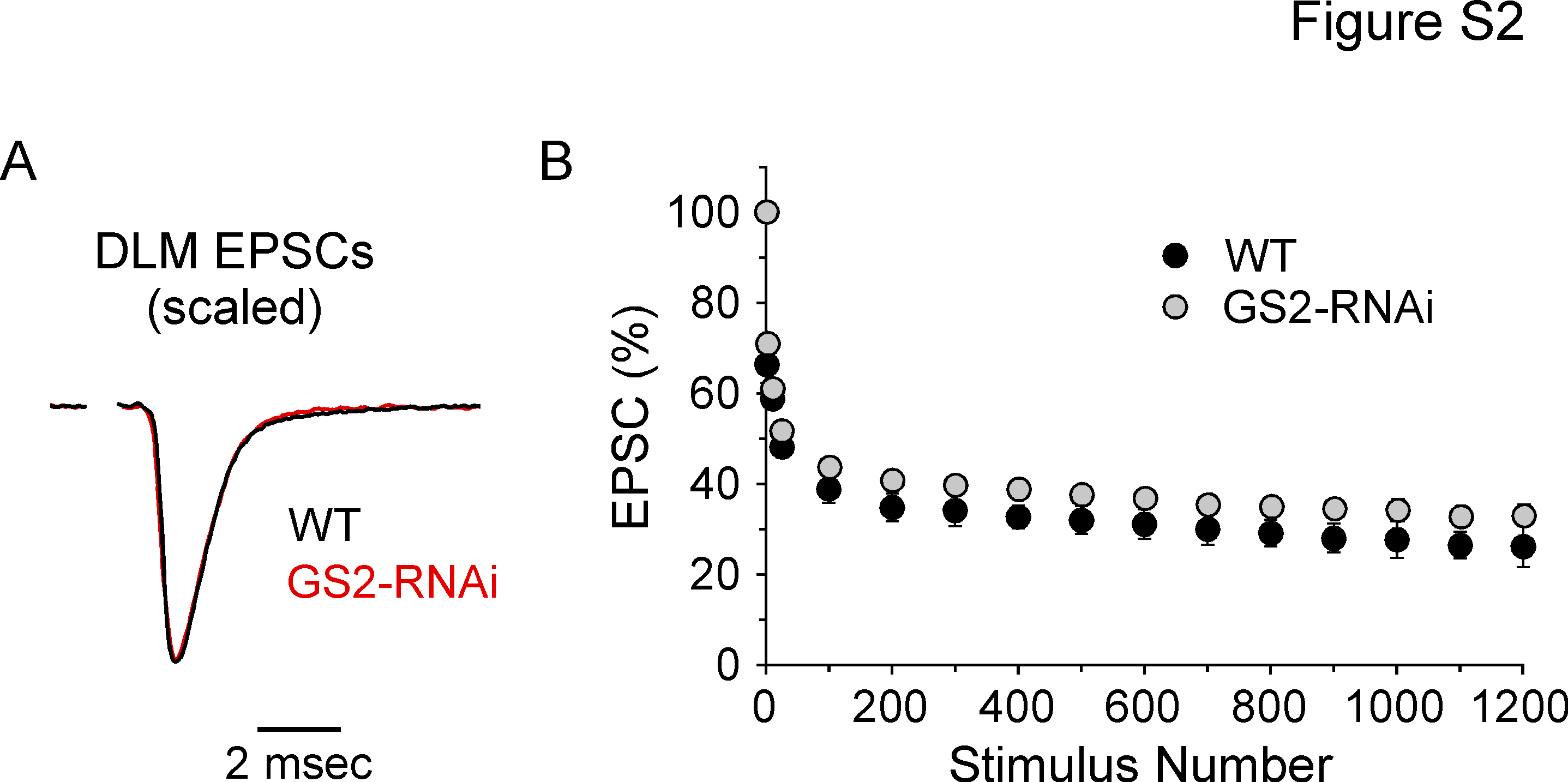

Supplement: S2 Fig — Two-electrode voltage-clamp recordings of synaptic currents from DLM neuromuscular synapses. (A) Scaled and superimposed single EPSCs indicate similar EPSC waveforms at wild-type (WT) and Gs2-RNAi KD synapses. For the Gs2-RNAi KD, the pan-glial repo-GAL4 driver was used to express the UAS-Gs2-RNAi transgene. The initial EPSC amplitudes for WT and GS2-RNAi KD were 1.88 ± 0.15 μA (n = 4) and 1.71 ± 0.18 μA (n = 4), respectively, and were not significantly different (p = 0.28). (B) Gs2-RNAi KD synapses exhibit wild-type short-term depression during sustained train stimulation at 20 Hz. Peak EPSC amplitudes were normalized to the initial amplitude and plotted as a function of stimulus number. After 60 seconds of train stimulation, the EPSC amplitudes for WT and Gs2-RNAi KD were reduced to 26.0 ± 4.56% (n = 4) and 33.0 ± 2.46% (n = 4), respectively. These values were not significantly different (p = 0.07), although there may be slightly less synaptic depression at Gs2-RNAi KD synapses. Error bars represent the S.E.M. (TIF) [file pone.0129957.s002.tif]

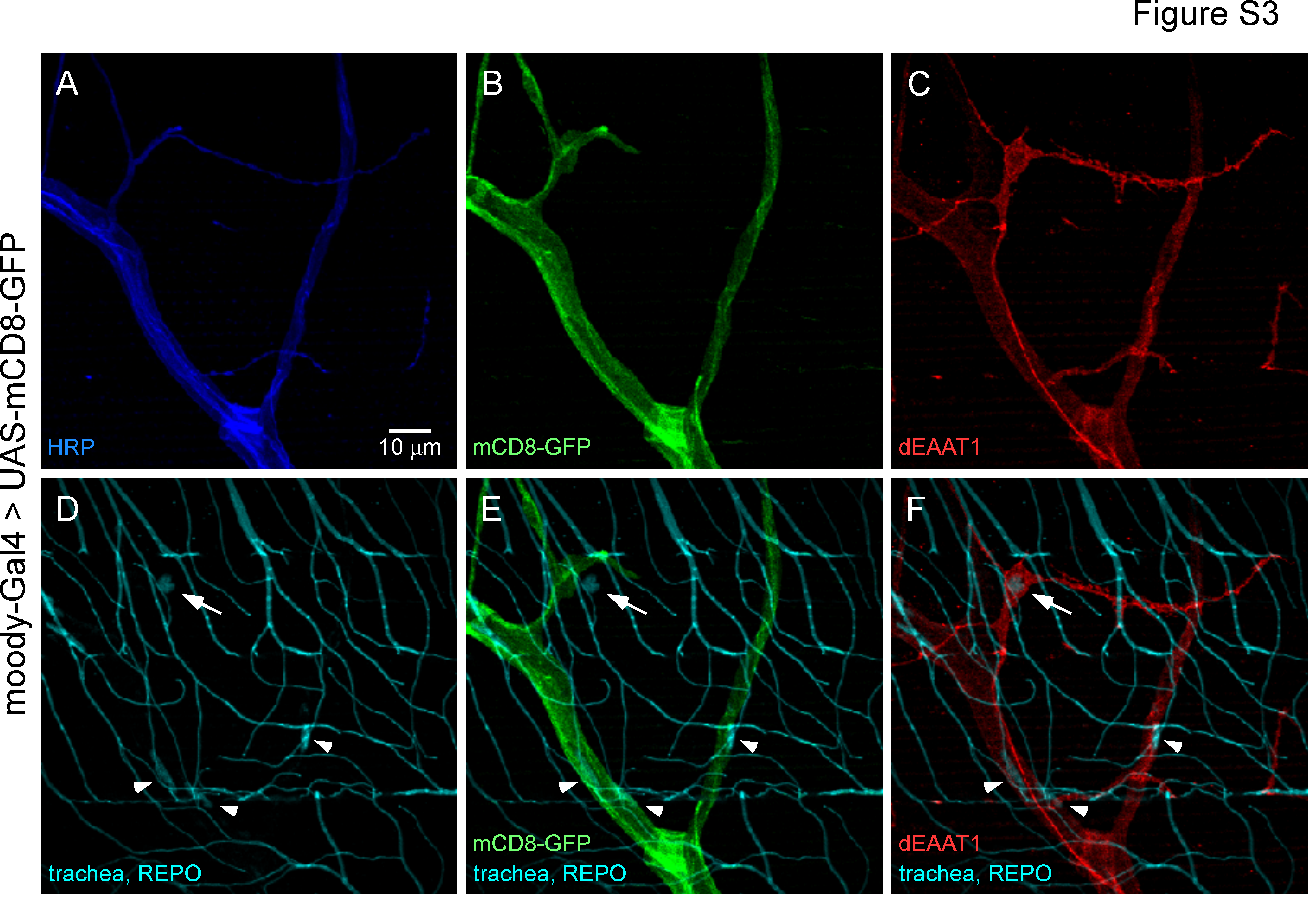

Supplement: S3 Fig — Confocal immunofluorescence and native GFP fluorescence images of DLM neuromuscular synapses. Experiments to examine REPO expression in PPG using the available mouse monoclonal anti-REPO antibody could not utilize the mouse monoclonal anti-GS2 antibody to selectively label PPG as in Fig 4. Rather, an alternative strategy employed moody-GAL4 to drive mCD8-GFP expression and mark ensheathing subperineurial glia as in Fig 2. After labeling with the rabbit polyclonal anti-dEAAT1 antibody, PPG can be distinguished as those glia expressing dEAAT1 but not GFP (A-C). Labeling with the anti-REPO antibody was imaged in the same channel with autofluorescence from the tracheal system (D-F, trachea, REPO). Anti-REPO marked the nuclei of ensheathing glia (arrowheads) as well as PPG (arrow). Note that these results are consistent with loss of GS2 signal in PPG after GS2 KD using the repo-Gal4 driver (Fig 3C–3L). (TIF) [file pone.0129957.s003.tif]
